# SARS-CoV-2 genomic contextual data harmonization: recommendations from a mixed methods analysis of COVID-19 case report forms across Canada

**DOI:** 10.1186/s13690-025-01604-5

**Published:** 2025-04-30

**Authors:** Rhiannon Cameron, Sarah Savić Kallesøe, Emma J. Griffiths, Damion Dooley, Aishwarya Sridhar, Anoosha Sehar, Lauren C. Tindale, William W.L. Hsiao

**Affiliations:** 1 Faculty of Health Sciences, Simon Fraser University, Burnaby, BC Canada; 2 Bioinformatics Graduate Program, University of British Columbia, Vancouver, BC Canada; 3 Department of Pathology & Laboratory Medicine, University of British Columbia, Vancouver, BC Canada; 4 Department of Molecular Biology and Biochemistry, Simon Fraser University, Burnaby, BC Canada

**Keywords:** COVID-19, SARS-CoV-2, Metadata, Data collection, Data curation, Public health, Correlation of data, Canada

## Abstract

**Background:**

The timely sharing of public health information is critical during a pandemic and is an obstacle that Canada has yet to fully address. During the COVID-19 pandemic, sequencing of the SARS-CoV-2 genome enhanced our understanding of transmission patterns, aided in identifying variants of concern, and supported the development and evaluation of diagnostic tests and vaccines. The Canadian national response faced challenges in aggregating genomic contextual data and carrying out integrated analysis across regions partly due to disparities in COVID-19 case report forms used to capture epidemiological and clinical data that accompanies SARS-CoV-2 sequence data. Such variations delay data integration and make consistent analysis difficult or impossible. The objective of this work was to understand what information was being collected from COVID-19 case report forms used across Canada and identify potential contextual data harmonization issues and solutions.

**Methods:**

Provincial/territorial/national Canadian COVID-19 case report forms were subjected to field-by-field comparisons to identify variations in data categorization, structures, formats, types, granularity, ambiguity, and questions asked. Federal epidemiologists were consulted to substantiate the results.

**Results:**

Data harmonization issues and common data elements were identified. We make recommendations for better national coordination, integrated databases, and data harmonization tools.

**Conclusion:**

This report compares data elements of the various case report forms used across Canada to identify overlaps and differences in the collection method of COVID-19 case information, while also highlighting data harmonization complications and potential solutions. Identifying available data elements will better guide COVID-19 surveillance and research.

**Supplementary Information:**

The online version contains supplementary material available at 10.1186/s13690-025-01604-5.

**Table:** 

**Text box 1. Contributions to the literature**
• Highlighting the specific data harmonization challenges that can and have emerged from the use of different collection forms is beneficial for the purpose of generating interoperable and comparable datasets, especially at the national scale.
• Knowing what data elements are commonly collected informs researchers and epidemiologists of what is and is not available to them for the design of infectious pathogen surveillance and/or research questions.
• Changing existing systems for collecting health data is expensive and time consuming; analyzing data collection forms is an immediate action that can be used for developing interim solutions and informing future changes that improve data sharing.

## Background

Canada faces challenges in data comparison and integration across regions due to disparities in how questions and data are structured across the case report forms used to capture contextual data. Case report forms are questionnaires often used in public health investigations and surveillance activities to capture epidemiological information regarding an ill individual. This data can then be used to enrich genomic data during pathogen sampling. Contextual data is information that allows us to better understand the environment and circumstances surrounding sequence data, e.g. clinical case information, epidemiological data, laboratory conditions, methods, and genomic annotations. While the genomic sequence data tells us the genetic code, the contextual data tells the essential story of who, why, what, and how. Contextual data variations hinder consistent genomic analysis, limiting epidemiologists’ ability to perform large-scale data discovery and aggregation [1]. A crucial element of the Coronavirus Disease 2019 (COVID-19) genomic response is acquiring harmonized case data in order to construct a deeper understanding of the spread of severe acute respiratory syndrome coronavirus 2 (SARS-CoV-2) and the efficacy of public health interventions.

### The Canadian health care system

Canada’s health care system is decentralized, meaning that the ten provinces and three territories independently administer separate health care systems within their jurisdictions to provide care to their residents and are the sole custodians of the health data [2, 3]. Together these systems interlock to create a universal, single-payer health care system. While this structure offers advantages, such as allowing provinces/territories to develop methods of delivering healthcare that is appropriate for their population and geographical region, a salient vulnerability is the lack of a single, overarching authority to coordinate the health care data management practice. Provinces and territories are not legally obligated to follow federal recommendations pertaining to health care or health data sharing [4], they also maintain their autonomy when it comes to regulating the collection of health care information [5]. Within a province or territory, there may be regional health authorities, or other front-line public health organizations, that have their own processes for health information data management [3].

### National level data collection

Genomic sequencing of the SARS-CoV-2 virus around the world has enabled tracking of the viruses, identification of variants, development of diagnostic assays, vaccines, and therapeutics [6–8]. Large datasets allow the nation to conduct analyses with national coverage, which is important for the health of all Canadians. National level data also helps the federal jurisdiction determine when it is necessary to close borders or call a national emergency. However, the lack of coordinated data sharing practice across the numerous independent public health authorities in Canada have resulted in delayed access and exchange of COVID-19 genomic and epidemiological information and reduced data quality due to variability in data streams. As a consequence of a lack of data standards, Canadian COVID-19 case report forms are designed independently by provincial/territorial health authorities based on the perceived needs of each jurisdiction. Provincial health laboratories do not necessarily standardize their case report forms for their jurisdictions either. While a national case report form was made available to use, there are many reasons why a province/territory may have chosen not to use it: absence of form questions necessary for jurisdiction-specific objectives, a lack of capacity to update active forms and data systems, and barriers to disseminating the form to data collectors.

### Provincial/territorial data collection

**Fig. 1:**
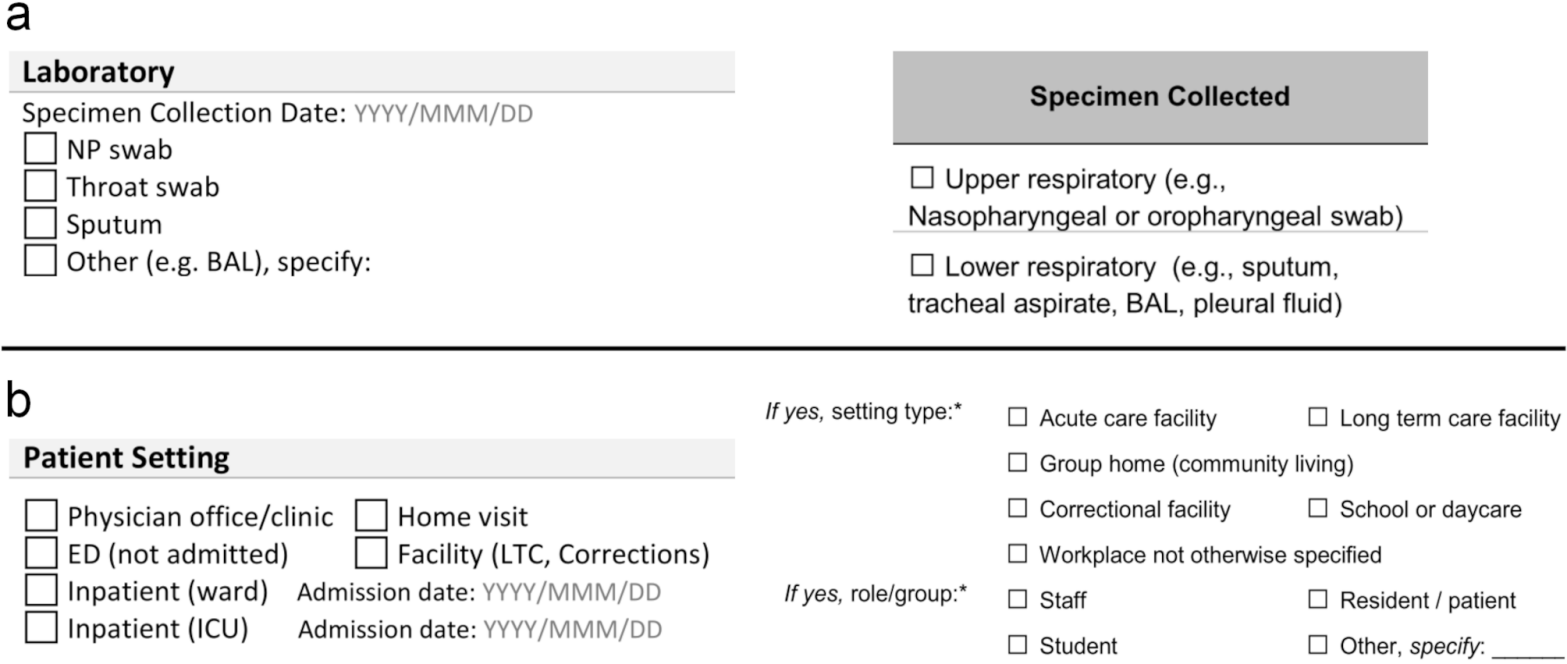
Differences in how information is collected across case report forms. (**a**) Excerpts of “Specimen Collection” information from NWT (left) and BC (right). In this example, the different forms use abbreviations and encode specimen information at different levels of granularity. The NWT “Laboratory” section asks for “Specimen Collection Date: YYYY/MMM/DD”, along with checkbox options for “NP swab”, “Throat swab”, “Sputum”, and “Other (e.g. BAL), specify:”. The BC “Laboratory” subsection “Specimen Collected” asks for checkbox entry for “Upper respiratory (e.g., Nasopharyngeal or oropharyngeal swab)” and “Lower respiratory (e.g., sputum, tracheal aspirate, BAL, pleural fluid). (**b**) Excerpts of “Patient Setting” information from NWT (left) and BC (right). The NWT “Patient Setting” section requests checkbox entry for “Physician office/clinic”, “Home visit”, “ED (not admitted)”, “Facility (LTC, Corrections)”, and then lists checkboxes and YYYY/MMM/DD “Admission date” data for “Inpatient (ward)” and “Inpatient (ICU)”. The BC “Exposures” subsection for exposures that may have occurred 14 days prior to symptom onset request checkbox confirmation for settings of “Acute care facility”, “Long form care facility”, “Group home (community living)”, “Correctional facility”, “School or daycare”, “Workplace not otherwise specified”; along with the role/group relation via checkbox confirmation for “Staff”, “Resident / patient”, “Student”, and “Other, specify”. In this example, it can be observed that different questions are being asked using the same field, e.g., “LTC” and “Long term care facility”. Figure adapted from “Comparison and analysis of Canadian public health SARS-CoV-2 case report forms” [23]

**Fig. 2:**
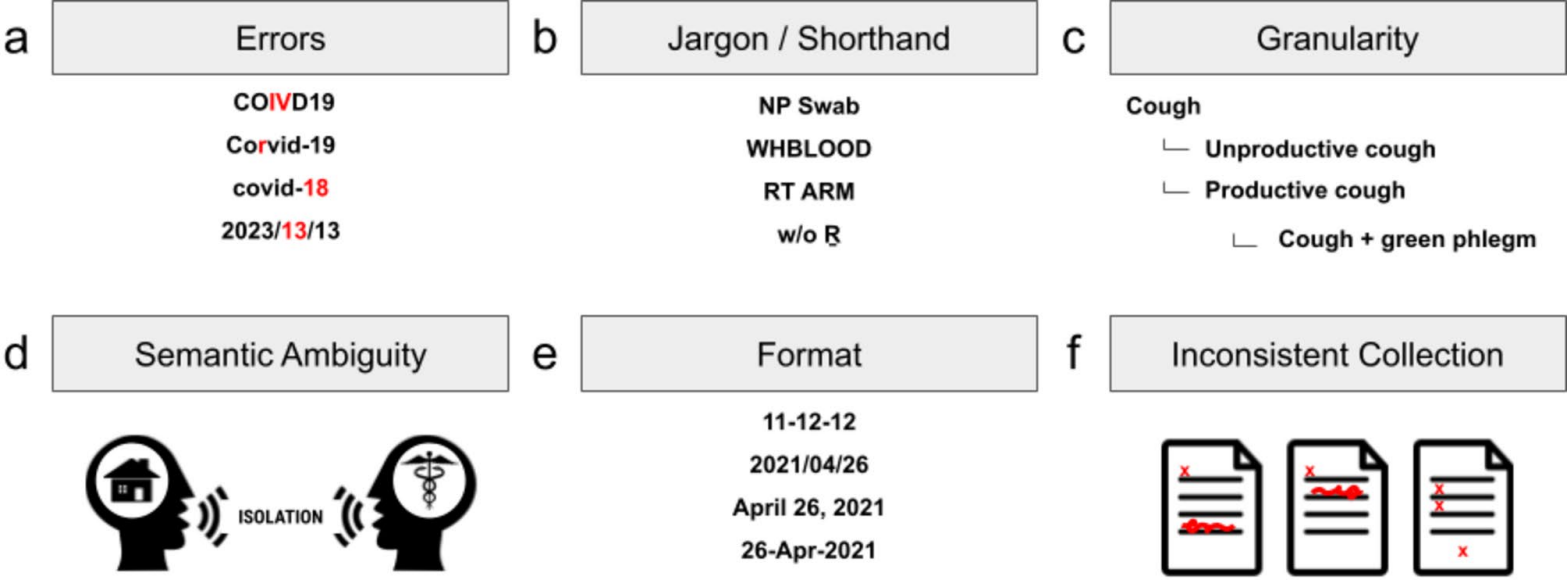
Examples of common data curation challenges. (**a**) Input errors. (**b**) the use of jargon or shorthand that isn’t necessarily known outside the data collectors. (**c**) Data collected at different granularities, which can cause issues if data systems and curators do not know the hierarchical relationships. (**d**) semantic ambiguity; the image shows two people using the term “isolation” while one envisions at home (depicted by a house) and the other in a medical facility (depicted by the Caduceus staff). (**e**) Different date formats. (**f**) Inconsistent data collection; image shows forms with different fields filled out and/or the same fields filled out differently

While the content of provincial/territorial forms are similar, the information is often encoded differently. There can be differences in how the information is structured, the kinds of questions being asked, and in the terminology being used that may cause discrepancies in downstream data (Fig. 1). Additionally, front line data collectors (e.g. hospitals, physician’s offices, clinics, etc.) do not tend to use the case report forms, which focus on the objectives of provincial epidemiologists, and instead work with the electronic systems, forms, and tools fit for their priorities. Front line data collectors transform their case information to fit their region’s case report form when submitting sequence samples, adding an additional layer of variability to contextual data flow. These accumulative differences render data comparison and integration more burdensome and error-prone by causing data corruption or failures during merges and uploads, requiring manual intervention when software design does not anticipate the disparities. Consequently, when data needs to be integrated for inter-jurisdictional analyses (e.g., inter-provincial outbreak investigations and surveillance), the data must be restructured and cleaned - a process which is time-consuming and labor intensive. If the meaning of information is not clear to curators (Fig. 2), they will need to go back to the data providers and ask clarifying questions. While this is feasible for the contextual data of a small number of sequences, these issues become extremely burdensome when dealing with 100,000s of sequences.

### Data harmonization

Both contextual and sequence data have issues with variability. Genomic data has widely accepted and used formats available for storing genetic variation data (e.g., FASTQ [9], VCF [10], and VRS [11]) making it less variable and usually not the primary source of heterogeneity. However, differences in protocols and technology can result in differences in genomics sequence data, adding to the burden of contextual data to track methodology. For example, using different sequence *de novo* assemblers can artificially exceed outbreak thresholds, which does not happen when the data is normalized with standardized whole-genome sequencing protocols [12]. Harmonizing both genomic and contextual data makes epidemiological outbreak investigations more efficient and effective.

While Canada has produced reliable genomic data for tracking and controlling infectious disease, contextual data (largely from case report forms) are needed to interpret the sequence data and address broader epidemiology questions. Thus, it is important that contextual data is shared in a timely manner, but variations slow down efforts to perform large-scale, consistent analyses, and the intra-provincial/territorial/agency nature of how health data is collected within Canada makes it difficult to apply solutions at the case report form stage of data collection. A viable, short-term alternative to addressing the inconsistencies of data sharing in Canada, specific to COVID-19, is to investigate the current methods of data collection to implement data harmonization solutions. Data harmonization reconciles differences between data streams, ensuring interoperability across datasets by standardizing fields, terms, and formats. Such an investigation into the variability of COVID-19 genomic contextual data would identify data sharing gaps that prevent more robust epidemiological, biomedical, and genomic analyses. Employing data harmonization tools would help address these gaps and help provide the best available evidence for governments across the country to guide public health action.

### The Canadian COVID genomics network

The lead authors of this study had experience working on data curation and harmonization delays with Canadian public health laboratories before this work. We also were actively involved in the curation of SARS-CoV-2 contextual data submitted to the national database, during the COVID-19 pandemic, as part of the Canadian COVID Genomics Network (CanCOGeN) VirusSeq initiative. The CanCOGeN initiative is a pan-Canadian partnership among academia; private sector; and regional, provincial/territorial, and federal governments to obtain and coordinate SARS-CoV-2 virus and patient host genomic sequence data as well as clinical/epidemiological contextual information. We were and are presently involved with Public Health Alliance for Genomic Epidemiology (PHA4GE) [13] which provided us insights into what contextual data sharing challenges were going on in other countries. The original goal of the CanCOGeN VirusSeq initiative was the sequencing of 150,000 SARS-CoV-2 positive patient samples, but in January 2025 the number of viral genomes sampled surpassed 619,000 [14] all of which have been harmonized to the SARS-CoV-2 data specification which was published in August 2020 [15].

### Case report form analysis

While developing the CanCOGeN viral contextual data specification for national surveillance, we found that jurisdictions were often unaware of what one another was doing. These discrepancies motivated our investigation and analysis of publicly available Canadian case report forms in order to propose new data standards that improve the ease, quality, and capabilities of genomic health data management. This analysis compares data elements of the various COVID-19 case report forms used across Canadian jurisdictions to understand what kinds of information are collected, how they are encoded, and how they may cause barriers to data harmonization. We also observed what elements are consistently available and thus should be prioritized to facilitate the harmonization of SARS-CoV-2 contextual information.

## Methods

This work utilizes exploratory descriptive research (EDR) methodology, focusing on understanding differences and commonalities across case report forms that collect epidemiological information. EDR studies facilitate exploratory and interpretive qualitative research to describe phenomena of interest that cannot be performed by conventional design methods [16]; in this case looking at the distribution and quality of data elements generated by content analysis of documents. Canadian federal, provincial, and territorial case report forms that target confirmed or presumptive SARS-CoV-2 infection cases were obtained electronically between 2020-03-03 and 2020-04-28 via open-access public health websites (Table 1). The most up-to-date versions of case report forms were obtained during the first few months of the COVID-19 pandemic and thus may not reflect changes to provincial, territorial, or national forms after June 1st, 2020. Provinces and territories that required the use of multiple forms are referenced when one or more of said forms utilized the data element/value of concern. Provinces and territories are abbreviated as follows: Alberta (AB), British Columbia (BC), Manitoba (MB), New Brunswick (NB), Newfoundland and Labrador (NL), Nova Scotia (NS), Nunavut (NU), Northwest Territories (NWT), Ontario (ON), Prince Edward Island (PEI), Québec (QC), Saskatchewan (SK), and Yukon (YK). Provincial and territorial forms were not observed in jurisdictions that reported to be using the Public Health Agency of Canada (PHAC) national case report form; namely, AB, NL, NS, NU, PEI, SK, and YK. French data items were directly translated by a research member with over 9 years’ experience in studying written and oral French (eight of which were immersion schooling). Fields with ambiguous meaning were initially paraphrased via Google Translate™ [17] before cross-checking against other non-COVID-19 case report forms or regional health documents that were available in both English and French. Any data elements that remained ambiguous were then confirmed by consulting with English-French bilingual medical doctors with working histories in both QC and BC.

**Table 1:** Canadian provinces/territories and their associated COVID-19 case report forms and version information

Province/Territory	Form	Version Number	Version Date (YYYY-MM-DD)
Alberta (AB) Newfoundland and Labrador (NL) Nova Scotia (NS) Nunavut (NU) Prince Edward Island (PEI) Saskatchewan (SK) Yukon (YK)	National - Public Health Agency of Canada (PHAC) Coronavirus Disease (COVID-19) Case Report Form	2	2020-03-03
British Columbia (BC)	BC COVID-19 Case Report Form		2020-04-20
Ontario (ON)	ON’s Severe Acute Respiratory Infection Case Report Form	7.0	2020-04-15
Québec (QC)	QC Coronavirus COVID-19 Déclaration Des Cas Confirmés Et Des Cas Cliniques De Covid-19	20-210-103 W	2020-04-28
	QC Coronavirus COVID-19 Questionnaire D’enquête Des Cas		2020-04-02
Manitoba (MB)	MB Coronavirus Disease 2019 (COVID-19) Investigation Case Form		2020-05-05
Northwest Territories (NWT)	NWT COVID-19 Report Form (Suspect Case/Person Under Investigation) - Part A		2020-04-27
	NWT COVID-19 Report Form (For All Cases) - Part B		2020-04-27
New Brunswick (NB)	NB COVID-19 Combined Referral and Lab Requisition Form	5	2020-04-09

Table adapted from “Comparison and analysis of Canadian public health SARS-CoV-2 case report forms” [20]. Copies of the case report forms are listed and available under the “Additional Materials” section of the manuscript

A mixed methods approach of qualitative content analysis and quantitative occurrence frequencies was performed on case report form data fields and their input values. Experienced Open Biological and Biomedical Ontology Foundry [18, 19] ontology data curators qualitatively mapped all provincial/territorial case report form data fields to the national form before a secondary review across all forms was performed to verify field mappings/counts. Ontologies are a form of structured, controlled vocabulary that use polyhierarchies and logical relationships to enable complex querying. Ontology curators are data professionals that ensure an ontology is accurate, consistent, and logically sound. When a data field could not be matched to the national form the newly identified field was added to the reference column and re-evaluated against all case report forms. On final review, input values were recorded as Boolean (true/false), free text, or as an enumerated list and were captured in the format that they appeared on the form. All fields and terms were investigated for their similarity in meaning and their differences in categorical organization, semantics, structure/format, and level of granularity. Comparisons were performed manually, occasionally requiring an inference of meaning from surrounding information due to a lack of a formalized unanimous schema or accessible data dictionaries. Imperfect matches were further analyzed for how their variations impede data harmonization. Data collection and processing was performed using Google Sheets™ [20].

All data elements were evaluated manually by ontology data curators for potential syntactic and/or semantic ambiguity, which is when a word can have multiple meanings that vary depending on the context [21]. Curation was performed by examining each instance of a data value against thesauruses; dictionaries; ontologies, encyclopedias, and usage examples identified on the world wide web. Categories and terms were evaluated to be exact matches (words deemed identical, including those with alternate spelling), synonyms (exact, narrow, or broad), or completely different values. Granular terms that could be classified under a broader umbrella synonym were permitted for counts of said broad synonyms, e.g., allowing “productive cough” to be classified as a “cough” for comparison with forms for which that was the highest level of granularity. Data values that contained more than one term (e.g., “Irritability/Confusion”) were analyzed in two different methodologies: (1) permitted as counts for the narrow use of the original terms (e.g., “Irritability” as well as “Confusion”) independent of one another, and (2) considered in the broadest use (e.g., “Irritability” counting towards “Irritability/Confusion” but not vice versa). To obtain frequency counts, raw data values were curated into clusters and given a label that appropriately encompassed all sub terms under approval of the curation team. From this information, we were able to highlight data harmonization and integration challenges that arise from the usage of distinct data collection instruments and then reached out to national epidemiologists via email to confirm that these challenges are factual.

## Results & discussion

Within Canada, there is no universal data collection form for SARS-CoV-2 infected individuals. Some provinces and territories use their own forms while others use a national form provided by PHAC, all of which were created for the reporting of confirmed and probable COVID-19 cases and to facilitate the identification of outbreaks. The national form was given significantly greater weight since seven out of thirteen provinces and territories were utilizing it at the time of this analysis (Table 1). The data elements of this study are primarily collected for applications in epidemiology and healthcare, but they can also be used to layer and combine with genomics results to use in public health intervention and surveillance (e.g., phylogenetic analyses, clinical manifestations of variants of concern, surveillance, etc.). The analysis informed what COVID-19 case-related information was available, the frequency at which they occurred; how the data was structured; and how data values needed to be carefully defined to capture data of varying granularity. We also reviewed and highlighted specific harmonization challenges that can and have emerged from the use of different collection forms for the purpose of generating interoperable and comparable datasets [22].

This information was critical in rapidly forming a pan-Canadian framework for public health emergency surveillance, enabling more efficient and accurate data sharing for the surveillance and analysis of SARS-CoV-2 and other pathogens. Our investigation focused on the critical moment of the early pandemic when SARS-CoV-2 data standards were not available. Countries around the world were, and still are, evaluating their genomic contextual data and looking internationally for standards as guidance. This analysis resulted in the publication of the case report form analysis CanCOGeN report [23] and the creation of a data standard (CanCOGeN VirusSeq) that is now being implemented internationally by other institutions and entities. The CanCOGeN VirusSeq standard was used as a foundation for the Canadian Genomics Research and Development Initiative for Antimicrobial Resistance (GRDI-AMR) specification [24, 25], the Alberta Microbiota Repository (AMBR) specification [26], and Canadian and International MPox specifications [27]. Additionally, the standard supported the development of the PHA4GE SARS-CoV-2, Wastewater Surveillance (WWS) [28], hAMRonization [29], and Quality Control tags [30], and Highly Pathogenic Avian Influenza (HPAI) [31] specifications. The PHA4GE SARS-CoV-2 specification has gone on to be utilized by SARS-CoV-2 sequencing initiatives in the USA, Australia, New Zealand, Africa, and Latin America [32].

### Common data elements

Data categories, elements and types that appeared in the majority or all Canadian case report forms were identified (Table 2). The focus of these results were on data explicit within a form, i.e., presented clearly within the text of the observed case report form, with any implicitly counted data flagged due to theoretical uncertainty over the match. For example, The NWT case report forms did not have an explicit *Hospitalized* field but did have a *Patient Setting* field with listed options for “inpatient (ward)” and “inpatient (ICU)”; from these inpatient options one could assume an individual was hospitalized at the risk adding false information. The most common fields and field categories used across all observed case report forms focused on the *Name*,* Date of Birth (DOB)*, *Phone Number*, *Gender*,* Symptom Onset Date*,* Symptoms* (often used synonymously with *Signs*), and *Pre-existing Conditions and Risk Factors* of the individual under observation (Table S1). Information that could facilitate the linkage of virus sequence contextual data with other datasets (e.g. Additional host sequence contextual data) include *Patient*,* Case*,* and Other Identifiers*; *Gender Field Values*; *Host Health State/Outcome*; *Host Health Status Details*; and *Host Resident Information* (Table S2). Along with assisting in general COVID-19 public health surveillance, this information permits the study of relationships between disease outcomes and host demographic information when appropriately linked. Categories collected to help determine COVID-19 manifestations and severity were determined to be *Signs and Symptoms*, *Pre-existing Conditions and Risk Factors*, and *Complications*. Clinical diagnoses found within these categories and deemed present in all case report forms can be found in Table S3. The data element *Symptom Onset Data* was also found to be present in all case report forms (Table S3), which is crucial since this information is vital for epidemiological inferences - such as quantifying incubation period (the window of time between initial infection and signs of illness) - and determining appropriate public health interventions.

**Table 2:** Universal SARS-CoV-2 Canadian case report form data items and the equivalent cancogen specification values

Generalized Data Field / Category	Generalized Picklist Term	CanCOGeN Equivalent	Ontology Identifier
Case Identifier		Case ID	GENEPIO:0100281
Name (First & Last)		N/A	N/A
Date of Birth		N/A	N/A
Phone Number		N/A	N/A
Gender		Host Gender	GENEPIO:0001395
Symptom Onset Date ^a^		Symptom Onset Date ^b^	GENEPIO:0001399
Signs & Symptoms		Signs and Symptoms	GENEPIO:0001400
	Cough	Cough	HP:0012735
	Fever ^c^	Fever Fever ( > = 38 °C)	HP:0001945 GENEPIO:0100066
	Headache	Headache	HP:0002315
	Sore Throat	Pharyngitis (Sore Throat)	HP:0025439
Pre-existing Conditions and Risk Factors		Pre-existing Conditions and Risk Factors	GENEPIO:0001401
	Cardiac Disease	Cardiac Disease	MONDO:0005267
	Diabetes	Diabetes Mellitus (diabetes)	HP:0000819
	Pregnancy	Pregnancy	NCIT: C25742
	Respiratory Disease	Respiratory Disorder	MONDO:0005087
Complications		Complications	GENEPIO:0001402
	Altered Mental Status	Altered Mental Status	HP:0011446
	Encephalitis	Encephalitis (Brain Inflammation)	HP:0002383

^a^ Significant variation in the recommended date format across case report forms: DD/MM/YYYY, MM/DD/YYYY, YYYY/MM/DD, YYYY/MMM/DD, and Unspecified

^b^ ISO 8601 standard “YYYY-MM-DD”

^c^ Minimum temperature that defines a fever has some variation between forms or is not defined

Case report form data items have been generalized to a single label for this table. Not Applicable (N/A) values occur because they were not appropriate for privacy protections of user data at the national level but would be of value to local and provincial levels. Ontology identifiers can be looked up online for more data item information

The analysis revealed which case report form data elements were universal, common, and uncommon; how data was structured; the impact of structure on ease of comparison, and how data values needed to be carefully defined to capture data of varying granularity. Universal fields and values were deemed useful across jurisdictions and thus were prioritized for inclusion in CanCOGeN VirusSeq data standard (Table 2). Universal and common case report form fields were added to CanCOGeN specification and labelled as “required”, “optional”, or “not applicable” based on discussions with provincial/territorial and national collaborators (Table 3). Fields labelled “not applicable” were considered too identifiable by privacy officers and not included in the specification. This work also highlighted what data elements can cause downstream data harmonization issues for the national analysis of SARS-CoV-2 for public health surveillance and intervention.

**Table 3:** SARS-CoV-2 cancogen specification values, submission requirements, and the equivalent Canadian case report form data fields

Generalized Data Field	CanCOGeN Field Equivalent	Ontology Identifier	CanCOGeN Requirement
**Database Identifiers**			
Case Identifier	Case ID	GENEPIO:0100281	Required
**Host Information**			
Complications	Complications	GENEPIO:0001402	Optional
Date of Birth	Host Age ^a^	GENEPIO:0001392	Required
Gender	Host Gender	GENEPIO:0001395	Required
Host Health Outcome	Host Health Outcome	GENEPIO:0001390	Optional
Host Health State	Host Health State	GENEPIO:0001388	Optional
Host Health Status Details	Host Health Status Details	GENEPIO:0001389	Optional
Host Resident Information			N/A
Indigenous Identification			N/A ^b^
Name (First & Last)			N/A
Personal Health Number			N/A
Phone Number			N/A
Pre-existing Conditions and Risk Factors	Pre-Existing Conditions And Risk Factors	GENEPIO:0001401	Optional
Signs & Symptoms	Signs And Symptoms	GENEPIO:0001400	Optional
Symptom Onset Date	Symptom Onset Date	GENEPIO:0001399	Optional
**Host Exposure Information**			
Exposure Additional Information / History Details	Exposure Details	GENEPIO:0001431	Optional
Exposure Event	Exposure Event	GENEPIO:0001417	Optional
Exposure Setting	Exposure Setting	GENEPIO:0001428	Optional
Exposures - Close, Direct, & Indirect Contact	Exposure Contact Level	GENEPIO:0001418	Optional
Host Role	Host Role	GENEPIO:0001419	Optional
Location of Exposure - Country	Location Of Exposure Geo_loc Name (Country)	GENEPIO:0001410	Optional
Location of Exposure - Travel History	Travel History	GENEPIO:0001416	Optional
Location of Exposure - Most Recent Travel - Destination City	Destination Of Most Recent Travel (City)	GENEPIO:0001411	Optional
Location of Exposure - Most Recent Travel - Destination State/Province/Territory	Destination Of Most Recent Travel (State/Province/Territory)	GENEPIO:0001412	Optional
Location of Exposure - Most Recent Travel - Destination Country	Destination Of Most Recent Travel (Country)	GENEPIO:0001413	Optional
Location of Exposure - Most Recent Travel - Departure Date	Most Recent Travel Departure Date	GENEPIO:0001414	Optional
Location of Exposure - Most Recent Travel - Return Date	Most Recent Travel Return Date	GENEPIO:0001415	Optional

^a^ “Host Age” is not an exact match for “Date of Birth”; it was used in the specification because it is less identifiable than “Date of Birth” and the age value was what is needed for CanCOGeN objectives

^b^ This field was deemed of high importance but is not currently under the custodianship of CanCOGeN data collection

Case report form data items have been generalized to a single label for this table. Not Applicable (N/A) values occur because they were not appropriate for privacy protections of user data at the national level, but could be of value to local and provincial levels. Ontology identifiers can be looked up online for more information. This table includes only a subset of CanCOGeN specification fields; the comprehensive specification is available online

### Data harmonization challenges

The following section discusses theoretical data harmonization issues that emerge as a consequence of using different Canadian case collection forms. Data harmonization issues in categorization, structure/format, values, granularity, semantics, and the use of disparate questions, were identified in this analysis (Table 4).

**Table 4:** Examples of harmonization issues identified in the case report form analysis

Issue	Example
**Data Categorization**	“Risk Factors” could be presented as “Pre-Existing Conditions”, “Exposures”, both, and neither.
**Data Structure/Format**	“03/04/2021” date; unclear whether “3rd of April” or “4th of March”.
**Data Type**	Fever = “TRUE” or “FALSE” (i.e., ☐ ) Fever = ≥ 38 °C Fever = 102.5 °F
**Data Granularity**	The terms “cough”, “dry cough”, “productive cough”, or “new onset cough” are used in different forms. When combining data, treating all these terms as synonyms can result in the loss of pathological information.
**Semantic Ambiguity**	Does “Isolation” mean “Self-Isolation”, “Home Isolation”, and/or “Hospital Isolation”? Is “Negative Pressure” applicable?
**Disparate Questions**	Not all forms request Indigenous identification data. Engagement with First Nations health authorities inconsistent.

Table adapted from “Comparison and analysis of Canadian public health SARS-CoV-2 case report forms” [20]

### Semantic ambiguity

A non-trivial issue across case report forms was how the meaning of words can differ between them, resulting in *semantic ambiguity* when the data value of interest can correspond to meanings different than the one intended. An example of an ambiguous term that appeared on case collection forms was “Isolation”. Without explicit explanation, it was unclear to the data user whether this corresponds to “Self-Isolation” [33], “Home Isolation” [34], or “Hospital Isolation” [35], all of which are examples of terms that appear on other case report forms. And if a form did indicate “Hospital Isolation” did this mean that the patient was put into a private room, away from other patients, or put under “Negative Pressure” conditions where there is a minimum number of air exchanges per hour? For example, being unable to distinguish between “Home Isolation” and “Hospital Isolation” may have consequences for epidemiologists when modeling the spread of the disease, as transmission in these scenarios are significantly different. Analysts and decision makers must form their own assumptions on the meaning of terms in order to parse data, should these assumptions not correspond to those made by the data recorder, research conclusions and policy implementations may not reflect the ground truth. One way to mediate this risk is to provide case report form users and downstream data entry personnel with a controlled vocabulary that clearly conveys the intended meaning.

### Data categorization

Case report forms vary in the overarching *categories* they use to house their data fields, sometimes making the underlying data fields difficult to correlate and consequently integrate. For example, “Pre-existing conditions” are a patient’s medical conditions prior to the infection of interest while “risk factors” are variables associated with increased risk of infection and can encompass internal (e.g. “pre-existing conditions”), external (e.g. “travel exposure”), or a combination of both (e.g. the behavioral risk of “smoking”). Since “risk factors” can encompass both “pre-existing conditions” and “exposures”, forms vary in their implementation - making it more difficult to collect, curate, and correlate underlying risk assessment data, potentially confounding analyses of risk. An overarching category may also change the field’s interpretation. For example, “hypotension” was a term found on all forms except for the provinces of NB and MB, in some cases under “Signs & Symptoms” while in others it was found under “Pre-Existing Conditions & Risk Factors”. These are not equivalent as the former implies a new symptom onset that correlates with the diagnosis while the latter is something the patient experienced prior to diagnosis and thus may have nothing to do with the disease of concern. While it may seem easy enough to differentiate this information within a single case report form, it limits the ability of a data curator to be certain that “hypotension” under “clinical information” in one data set can reasonably be matched to “hypotension” as a “sign & symptom” in a data set that used a different collection device. Moreover, as data passes from one partnering agency to another, the original context and usage of the data elements may be lost when the data are transcoded.

### Data structure/format

*Data structures* encompass a collection of values, their specialized intra-data relationships, organization, and how these values can be altered and operated on. They are usually designed for a specific purpose such that the intended interpolation can be appropriately inferred from the results. Date formats are an example of data structure; to represent a date, we structure it as three values, day, a month, and a year, with a specific temporal hierarchy. A date structure is formatted such that it informs what the data values represent (e.g., “01” within the month positions is inferred as “January”) and their relationship to one another (e.g., a day belongs within a month within a year). By applying a uniquely formatted representation to data, we avoid ambiguity in its interpretation.

However, not all case collection forms are consistent in how they structure date formats, resulting in an issue known as *structural* or *syntactic ambiguity*. While many were very clear in their intended structure, the national form used more than one date format within the same document, while the province NB specified no format at all. This can lead to ambiguity and misinterpretations between day, month, and even year (Table 5). For example, the date ”03/04/21” can result in misinterpretations between day, month, and year; it is not clear whether the example is referring to March 4th, April 3rd, or even the 21st day of April/March in the year 2003/2004. Not being consistent within a single form puts greater reliance on data entry personnel to catch these inconsistencies and - in the case of unclear formatting - lead to incomplete data, cross-referencing investigations, or literal guesswork. At this time the Government of Canada has declared the national standard to be the YYYY-MM-DD or YYYY-MM ISO 8601 international standard [36, 37]. This is not a requirement that provinces/territories need conform to and Canada does still accept dates in alternate formats. The misinterpretation of data formats on collection forms has the potential to cause significant problems in downstream data analysis, especially during the COVID-19 pandemic when getting epidemiological data analyzed is time-sensitive and misrepresentations of sampling dates have serious implications.

**Table 5:** Examples of structure variations date formats and symptom granularity used in Canadian case report forms

Case Report Form	Date Format	Data Granularity
**National** ^a^	DD/MM/YYYY MM/DD/YYYY	Cough
**BC**	YYYY/MM/DD	Cough
**MB**	YYYY-MM-DD	Cough, Dry; Cough, Productive
**NB**	*Free Text*	New onset/exacerbation of chronic cough
**NWT**	YYYY/MMM/DD	Cough
**ON**	DD/MM/YYYY	Cough
**QC**	YYYY/MM/DD	Cough

^a^ The following provinces/territories were utilizing the Interim National Case Report From at the time of analysis: AB, NL, NS, NU, PEI, SK, and YK

Date Format values: day (D), month (M), and year (Y). Provinces/Territories: Alberta (AB), British Columbia (BC), Ontario (ON), Québec (QC), Manitoba (MB), New Brunswick (NB), Newfoundland and Labrador (NL), Nova Scotia (NS), Nunavut (NU), Northwest Territories (NWT), Prince Edward Island (PEI), Saskatchewan (SK), Yukon (YK). Table adapted from “Comparison and analysis of Canadian public health SARS-CoV-2 case report forms” [20]

### Data types

Another issue that can add to data processing time is when the same or similar data fields have differences in *value types* between forms, resulting in data string variations that may not be easily compared and require different levels of process. For example, where one form may offer a Boolean (True/False) value in response to whether a case has a “fever” (i.e., “Yes/No”), another form may ask for the highest temperature recorded (Table 6). The latter may have no declared data structure informing the user whether temperature should be written as a string of characters or a number and whether it should be in Celsius or Fahrenheit. And if a data curator, who was not the data recorder, is presented with a checkbox, will an “x” (☒) be interpreted as TRUE like a checkmark (☑), or will the data curator infer a negative context and input FALSE? Comparison of dissimilar data types presents problems for computer-based analysis where information recorded differs from what the software is written to handle, causing data corruption, systems crashes, or unintentional transformations (e.g., entry of “Yes” into a field expecting a number, since a number was not received it returns “False” which a downstream user may assume was intentionally entered to convey “No”).

**Table 6:** Examples of data type variations when collecting “fever” information via Canadian case report forms

Case Report Form	Question	Input	Data Type / Information
**National** ^a^	Fever (≥ 38 °C)	☐ Yes ☐ No ☐ Unknown ☐ Not asked/assessed	TRUE/FALSE for fevers greater than or equal to 38 Celsius, missing value options
**BC**	Fever	☐ Yes ☐ No ☐ Asked but Unknown ☐ Declined to Answer ☐ Not Assessed	TRUE/FALSE or missing value options
	If yes, specify the highest temperature recorded:	____ °C	Free text; may be words or numbers
**MB**	Fever (> 38 °C)	☐	TRUE/FALSE only for fevers greater than 38 Celsius
**NB**	Fever/chills	☐	TRUE/FALSE for Fever and/or chills. Unless “Fever” is circled, data is unspecified as to whether a fever occurred
**NWT**	Fever	☐	TRUE/FALSE
	Temperature if known:		Free text; may be words or numbers, Celsius or Fahrenheit not specified
**ON**	Fever (≥ 38 °C)	☐	TRUE/FALSE for fevers greater than or equal to 38 Celsius
**QC**	Fever (≥ 38 °C)	☐ Yes ☐ No ☐ Unknown	TRUE/FALSE for fevers greater than or equal to 38 Celsius, missing value option

^a^ The following provinces/territories were utilizing the Interim National Case Report Form at the time of analysis: AB, NL, NS, NU, PEI, SK, and YK

Demonstrates the varying data types and information that can be collected across case report forms, many of which are similar but not exact. Temperature recordings may have additional context (e.g., BC this would be the highest recording if multiple measurements were taken), be a specific number when known (BC and NWT), be taken in different temperature scales (NWT could be recorded in Fahrenheit or Celsius while all others are in Celsius), and for some the definition of “Fever” vary (National, ON, and QC would consider “38°C” a fever while MB would not)

### Data granularity

A recurring complication in comparing data across case report forms was variation in granularity. In this context, granularity refers to the level of detail of a data element and how it is subdivided. Depth of analyses become limited when data collection sources contain variation that differentiates descriptors such that it can be difficult to match them to a common term. For example, “cough” as compared to “dry cough” [38], “productive cough” [38], or “new onset/exacerbation of chronic cough” as this differentiation in descriptors can result in inappropriate mappings and/or a loss of pathology information (Table 4). The inability for a pathologist to differentiate between dry and productive coughs can impact how respiratory diseases are defined and differentiated. Additionally, sometimes terms are grouped together without clear instruction or demarcation. Hypothetically, the data collector may indicate it to be “True” a case experienced “Nausea/Vomiting” because the patient had been nauseated. Downstream data entry/analysis personnel could interpret “Nausea/Vomiting” as a data point towards “Vomiting” when no vomiting had ever occurred, associating a false sign or symptom with a disease while also experiencing a loss of the intended “Nausea” data point. Multiple concepts in the same field create uncertainty (does “Nausea/Vomiting” indicate “Nausea”, “Vomiting”, or both? ) while also making it hard to fit data with other datasets where the concepts are in separate fields.

### Disparate questions

The presence of partially aligned but non-identical questions present another barrier to data normalization. Increasing the homogeneity of questions increases the capacity of investigators to perform detailed, large-scale analyses. For example, question disparity presents issues in the collection and analysis of demographic information. Forms may inquire whether a patient identifies as “First Nations”, “Inuit”, or “Métis”, and/or whether a patient resides on a reserve, or the form may not request any patient Indigenous identification data at all (Table 7). Because of this disparity, questions may be removed or severely limited when analyzing large combined datasets where the data values have partial but not complete overlap of meaning; for example, “lives on reserve” (whether the individual resides in a location with “reserve status” [39]) and “identifies as Indigenous” (self-determined Indigenous identification) are not equivalent.

We also identified questions with no overlap between case report forms. QC was the only province/territory form to inquire whether a patient experienced “pregnancy complications” or whether the patient was a worker exposed to direct customer contact. Similarly, NB was the only province/territory to list “coryza” (acute inflammation of the nasal passage) under the assessment of symptoms. This does not imply that these questions are not important to ask, but rather their value is lessened since they appear infrequently during the data collection process. One could argue that these questions are unique to the region and jurisdiction collecting them, however we could not identify any instances where this appeared to be the case. It is also reasonable to assume that other jurisdictions chose not to include these fields/values to limit the size of their case report form. There was no strict limit on case report form length, but too many fields increase the burden of data entry on health care workers and patients– increasing the likelihood of some portions being missed or skipped. Case report form designers recognize that requesting too much of the form users may result in diminishing or negative returns on data quality and quantity. Some coordination across the nation could significantly reduce provincial/territorial inconsistencies, especially among high-priority descriptors.

**Table 7:** Indigenous identification data fields across Canadian case report forms

Case Report Form	Identify as Indigenous	First Nations Status	First Nations	Métis	Inuit	Combination ^a^
**National** ^b^	✔		✔	✔	✔	
**BC**	✓	✓	✓	✓	✓	✓
**MB**	✓	✓	✓	✓	✓	
**NB**						
**NWT**						
**ON**	✓		✓	✓	✓	
**QC** ^**c**^	✓		✓		✓	

^a^ Options for “First Nations and Inuit”, “First Nations and Métis”, “First Nations, Inuit and Métis”, or “Inuit and Métis”

^b^ The following provinces/territories were utilizing the Interim National Case Report From at the time of analysis: AB, NL, NS, NU, PEI, SK, and YK

^**c**^ Only available on Québec form “QC Coronavirus COVID-19 Questionnaire D’enquête Des Cas” 2020-04-02

Provinces/Territories: Alberta (AB), British Columbia (BC), Ontario (ON), Québec (QC), Manitoba (MB), New Brunswick (NB), Newfoundland and Labrador (NL), Nova Scotia (NS), Nunavut (NU), Northwest Territories (NWT), Prince Edward Island (PEI), Saskatchewan (SK), Yukon (YK). Table adapted from “Comparison and analysis of Canadian public health SARS-CoV-2 case report forms” [20]

### Indigenous identification data

Eleven of the thirteen Canadian case report forms were found to collect up to four categories of identification data pertaining to Indigenous peoples in Canada. These categories include *First Nations Status*,* Identify as Indigenous*, *Indigenous Heritage*, and *Reservation/Community* information. Indigenous identification (regardless of community designation) data collection on case report forms is represented in Table 7. Collecting this type of information is important as it provides a means to highlight systemic inequalities impacting Indigenous populations, supporting positive interventions and policy change.

*First Nations Status* is a distinct legal status available to Indigenous peoples in Canada who qualify for the criteria [40]. The process of being legally recognized as having *First Nations Status* can be laborious and difficult, often resulting in many *First Nations* peoples not being granted this status [40]. Data regarding *First Nations Status* was only collected on the BC and MB forms. Both provinces included separate options to *Identify as Indigenous*, an important addition for acknowledging and acquiring data on *First Nations* who were ineligible for status. Capturing differences in status information is pertinent as it allows for the analysis of how status may impact health outcomes (e.g. via access to health and government services).

All case report forms that included the option to *Identify as Indigenous* also included some capacity to indicate *Indigenous Heritage* information. The *Indigenous Heritage* options were *First Nations*, *Métis*, and *Inuit*. That being said, the QC case report form did not include an option for *Métis* and the BC case report form provided additional explicit options for inputs of any combination of the aforementioned options; other forms did not restrict the selection of more than one option. Collecting this level of disaggregated data allows for a more diverse inequality analysis of potentially intersecting demographics [41]. The BC Office of the Human Rights Commissioner recommends the immediate collection of disaggregated demographic data in the area of health care [41]. In order to ensure that race-based data is being observed through the lens of reducing oppression and systemic racism, and not that of measuring race, custodianship of this data should be put within the hands of Indigenous organizations [41], however, this cannot be done if the appropriate Indigenous organization associated with the data cannot be identified.

Outside of the utility of Indigenous community demographic data for public health analysis, collecting Indigenous demographic data is important for the identification of the Indigenous nation and organization that are responsible for data custodianship under Indigenous data governance initiatives [42, 43]. The national, ON, and QC case report forms collected whether the patient resides on a *reserve*, while *Indigenous community* was collected on MB and NB - with the former only collecting this information if the patient was symptomatic. There is an important distinction to recognize between these terms; while a *reserve* is an *Indigenous community*, *reserves* are designated a specific *reserve status* that other *Indigenous communities* may not qualify for [39]. BC was the only province to implement the collection of *Indigenous organization information* (e.g. “Nazko First Nation”).

It is important for us to acknowledge that Indigenous identification data was not covered by the CanCOGeN VirusSeq specification. This was primarily due to the lack of appropriate and culturally sensitive data standards. The CanCOGeN metadata harmonization team is working towards identifying language that is appropriate for data capture with the assistance of the CanCOGeN Ethics and Governance Working Group, and consultation with Indigenous organizations will be a key part of further development.

### Recommendations

This work identified common Canadian COVID-19 case report form data elements and used them to build the foundation for the CanCOGeN VirusSeq data standard. We focused on case report forms from the early months of the pandemic, but future analyses could benefit from tracking the evolution of case report forms throughout an epidemiological event to observe how data items were refined and prioritized. Data harmonization challenges were identified in data categorization, structure, format, type, granularity, ambiguity, and questions asked. In order to address some of these challenges, we recommend pan-Canadian agency coordination to use an agreed upon standard, meaningful engagement with Indigenous peoples data governance boards, and the use of data harmonization tools.

Different institutions may have distinct form questions and data structures due to the unique circumstances and needs within their jurisdiction, potentially resulting in inconsistent and ambiguous information when merged with other datasets. Coordination between agencies across the nation to use an agreed upon standard when creating forms would make datasets more harmonizable from the start, significantly reducing inconsistencies at the point of data collection, data entry, and the linkage of contextual data with virus sequence data. In response to this need, CanCOGeN developed the CanCOGeN VirusSeq contextual data specification to facilitate the formation of well-structured, consistent contextual datasets from disparate sources across Canada [44]. Continuous data standard development also provides flexibility to meet provincial/territorial needs as they come up; otherwise, agencies are incentivized to create their own contextual data parameters when their needs are not met. We also recommend case reports form developers meaningfully engage with regional and national Indigenous peoples governance boards to determine what kinds of disaggregate data elements should be collected and to what granularity. At minimum, we recommend the following Indigenous demographic data elements be brought to the discussion: *Indigenous heritage* information, *First Nations status* (separated from heritage information), and *reservation/community/organization* information.

The nature of Canada’s decentralized health system means that data harmonization ends up being needed at both the provincial/territorial and national levels. Data generators at the province/territory level understand the biases and limitations of their data so they need to be the ones to transform and report it for sequence submissions. The further away someone is from the data source the more likely they will make mistakes, and misunderstandings can result in data transformation or complete data loss because they do not have access to individuals who are familiar enough with the data to resolve the issue. National level data submissions are also only a subset of what a province collects, which adds to the lack of context that may be necessary to resolve harmonization issues. Data wranglers at the national level will have to deal with issues from user input errors, submissions from inexperienced curators (e.g. staff turnover), and unexpected incompatibilities that arise from data flow system updates. Since data harmonization will inevitably happen at both levels, the harmonization of data flow will ultimately reduce the workload on provinces/territories and national laboratories. That being said, the national level is best positioned to collaborate and observe the “big picture” harmonization needs across provincial/territorial data submissions and thus should take on the burden of designing harmonization specifications and tools that can then be provided to the provincial/territorial levels to facilitate the transformation of their data for national submissions.

When a national specification is provided the data entities and allowable inputs need to be clearly defined to support lower-level harmonization. A key element will be an exchange-format framework that aligns specification elements with the requirements for relevant sequence repositories, creating a throughline from data collection to sequence submission. In the case of the CanCOGeN specification, we used the ontology-based standard as the foundation on which we encode cross references to data elements in databases such as the Canadian VirusSeq Portal [14], CNPHI (Canadian Network for Public Health Intelligence) [45], GISAID (Global Initiative on Sharing Avian Influenza Data) [46], and the NCBI (National Center for Biotechnology Information) BioSample [47]. Dynamic standards are preferred to fixed ones as they can adapt to unexpected harmonization challenges, new research questions, and user experience. Fixed standards tend to fall into disuse when they fail to anticipate current and future needs. There should be avenues for feedback from data generators to accommodate their data needs and implementation barriers as well as efficient and clear means of updating data generators when changes occur. It is helpful to make specification elements pathogen agnostic, when possible, that way both provincial and national data flow systems can reuse vetted data elements with which they are already familiar.

In lieu of asking provinces/territories to change their current SARS-CoV-2 case report form(s), as changing internal procedures can be difficult and time consuming, we recommend addressing the national data sharing inconsistencies by encouraging provinces and territories to use database-integrated or stand-alone data harmonization tools to improve data comparability and interoperability. One such tool, developed by CanCOGeN based on the aforementioned standard, is the DataHarmonizer [48]. The DataHarmonizer utilizes the flexible standardization of ontologies [18]; offers controlled vocabularies and minimal data standards, such as the CanCOGeN and PHA4GE COVID-19 specification; and minimizes data transformation by allowing customizable template imports while facilitating export to multiple genomic databases. All Canadian provinces/territories and the National Microbiology Laboratory each have their own DataHarmonizer installation to facilitate pan-Canadian SARS-CoV-2 harmonization. Publicly available DataHarmonizer specification templates are available in the “Pathogen Genomics Package” GitHub repository [49] and software improvements are in development that will allow users to create their own specifications using components from the CanCOGeN, PHA4GE, and other standards.

### Limitations

While many health regions used the form(s) agreed upon throughout an individual province/territory, some regions had agency or location-specific case report forms that did not correspond to the provincial/territorial forms utilized in this report. This analysis was also limited to the use of case report forms that were publicly accessible and available online, excluding theoretically private or non-electronically published forms. Consequently, the results were skewed towards publicly accessible, electronic copies of case report forms that were deemed most likely to be in use and thus this analysis was not inclusive of all case report forms utilized across Canada. It also did not look at previous versions of case report forms that may have been used during the pandemic, potentially missing data harmonization issues that could have impacted downstream SARS-CoV-2 datasets. Researchers were unable to locate official English translations of the French QC forms; it is possible that context was lost during unofficial translation. In the case of the QC results, positive hits were indicated if a field was identified on either form which may falsely inflate the commonality of the data fields collected. Additionally, due to the nature of qualitative analysis and the consequential impact of researchers on the interpretation of mappings, researchers outside this analysis may disagree with mappings and harmonization issue classifications; such disagreement further highlights the difficulty of data element interpretation and the potential for data harmonization complications.

## Conclusions

This COVID-19 case report form analysis helped structure the CanCOGeN data standard by identifying which genomic data parameters are commonly being collected, informing partner agencies of what was and was not available to them for the design of surveillance and/or research questions. The analysis also informed whether a data field should be required, recommended, or optional; how data was structured; and how data fields and values needed to be carefully defined to capture data values of varying granularity. Understanding where data harmonization challenges occur on a provincial/territorial level helps in the development of solutions that can be offered to all stakeholders without overstepping jurisdictional boundaries that can result from trying to resolve these issues at the data collection level. While this work was completed to facilitate inter-provincial/territorial data sharing under the SARS-CoV-2 national emergency, the lessons we have learned can be leveraged for the surveillance and analysis of other human pathogens.

## Electronic supplementary material

Below is the link to the electronic supplementary material.

Supplementary Material 1

## Data Availability

The datasets supporting the conclusions of this article are available in the “Canadian COVID-19 Case Report Form Analysis Files” Open Science Framework repository, 10.17605/OSF.IO/4UA8P.

## Abbreviations

AB: Alberta
AMBR: Alberta Microbiota Repository
BC: British Columbia
CanCOGeN: Canadian COVID-19 Genomics Network
CNPHI: Canadian Network for Public Health Intelligence
COVID-19: Coronavirus Disease 2019
EDR: Exploratory descriptive research
GISAID: Global Initiative on Sharing Avian Influenza Data
HPAI: Highly Pathogenic Avian Influenza
QC: Québec
MB: Manitoba
MPox: Monkeypox
NB: New Brunswick
NCBI: National Center for Biotechnology Information
NL: Newfoundland and Labrador
NS: Nova Scotia
NU: Nunavut
NWT: Northwest Territories
ON: Ontario
PEI: Prince Edward Island
PHA4GE: Public Health Alliance for Genomic Epidemiology
PHAC: Public Health Agency of Canada
SARS-CoV-2: Severe acute respiratory syndrome coronavirus 2
SK: Saskatchewan
USA: United States of America
WWS: Wastewater Surveillance
YK: Yukon
