# Supplementary material for: SARS-CoV-2 genomic contextual data harmonization: recommendations from a mixed methods analysis of COVID-19 case report forms across Canada
Source: Arch Public Health. 2025 Apr 30;83:117. doi: 10.1186/s13690-025-01604-5 (PMC12042453; doi:10.1186/s13690-025-01604-5)
Supplement: Supplementary file 1 — Supplementary Material 1 [file 13690_2025_1604_MOESM1_ESM.docx]

# Supplementary Tables:

**Table S1. Overview of the data fields and field categories commonly found in the Canadian case report forms.**

| **Case Information** | Case Report Form | | | | | | |
| --- | --- | --- | --- | --- | --- | --- | --- |
|  | **National** ^a^ | **BC** | **MB** | **NB** | **NWT** | **ON** | **QC** |
| Name (First & Last) | ✔ | ✓ | ✓ | ✓ | ✓ | ✓ | ✓ |
| Date of Birth **^b^** | ✔ | ✓ | ✓ | ✓ | ✓ | ✓ | ✓ |
| Phone Number | ✔ | ✓ | ✓ | ✓ | ✓ | ✓ | ✓ |
| Gender | ✔ | ✓ | ✓ | ✓ | ✓ | ✓ | ✓ |
| Symptom Onset Date **^b^** | ✔ | ✓ | ✓ | ✓ | ✓ | ✓ | ✓ |
| Symptoms | ✔ | ✓ | ✓ | ✓ | ✓ | ✓ | ✓ |
| Pre-existing Conditions and Risk Factors | ✔ | ✓ | ✓ | ✓ | ✓ | ✓ | ✓ **^c^** |
| ^a^ Applicable Provinces: AB, NL, NS, PEI, SK, YK.  ^b^ Date formats are not consistent across all forms.  **^c^** Only available on Québec form “QC Coronavirus COVID-19 Questionnaire D’enquête Des Cas” 2020-04-02. | | | | | | | |

Case report form data items have been generalized to a single label for this table. “Case information” includes data elements associated with the host/patient being observed/diagnosed/tested. Table adapted from “Comparison and analysis of Canadian public health SARS-CoV-2 case report forms” [[20]](https://paperpile.com/c/VHLwzB/HO8nH).

**Table S2.** **Overview of “General Case Information” data fields commonly found in the Canadian case report forms.**

| **General Case Information** | **Case Report Form** | | | | | | |
| --- | --- | --- | --- | --- | --- | --- | --- |
|  | **National** ^a^ | **BC** | **MB** | **NB** | **NWT** | **ON** | **QC** |
| **Patient, Case, and other Identifiers** | | | | | | | |
| Personal Health Number |  | ✓ | ✓ | ✓ | ✓ |  | ✓ |
| Case and/or Other Identifiers | ✔ | ✓ | ✓ |  |  | ✓ | ✓ **^c^** |
| **Gender Field Values** | | | | | | | |
| Female, Male | ✔ | ✓ | ✓ | ✓ | ✓ | ✓ | ✓ |
| Unknown | ✔ | ✓ | ✓ |  |  | ✓ |  |
| **Host Health State / Outcome** | | | | | | | |
| Symptomatic, Deceased | ✔ | ✓ | ✓ | ✓ | ✓ | ✓ | ✓ **^c^** |
| Asymptomatic | ✔ | ✓ | ✓ |  | ✓ | ✓ | ✓ **^c^** |
| **Host Health Status Details** | | | | | | | |
| Hospitalized | ✔ | ✓ | ✓ | ✓ | ✓ ^b^ | ✓ | ✓ **^c^** |
| ICU, ICU Start Date | ✔ | ✓ | ✓ |  | ✓ | ✓ | ✓ **^c^** |
| Date of Death  / Disposition Date | ✔ | ✓ | ✓ |  | ✓ | ✓ | ✓ **^c^** |
| **Host Resident Information** | | | | | | | |
| City | ✔ | ✓ | ✓ | ✓ | ✓ | ✓ | ✓ |
| Address, Postal Code | ✔ | ✓ | ✓ | ✓ |  | ✓ | ✓ |
| ^a^ Applicable Provinces: AB, NL, NS, PEI, SK, YK.  ^b^ Implied based on patient setting selection. **^c^** Only available on Québec form “QC Coronavirus COVID-19 Questionnaire D’enquête Des Cas” 2020-04-02. | | | | | | | |

Case report form data items have been generalized to a single label for this table. Table adapted from “Comparison and analysis of Canadian public health SARS-CoV-2 case report forms” [[20]](https://paperpile.com/c/VHLwzB/HO8nH).

**Table S3. Overview of “Clinical Diagnoses” data fields commonly found in the Canadian case report forms.**

| **Clinical Diagnoses** | **Case Report Form** | | | | | | |
| --- | --- | --- | --- | --- | --- | --- | --- |
|  | **National** ^a^ | **BC** | **MB** | **NB** | **NWT** | **ON** | **QC** |
| Symptom Onset Date ^b^ | ✔ | ✓ | ✓ | ✓ | ✓ | ✓ | ✓ |
| **Signs and Symptoms** | | | | | | | |
| Cough | ✔ | ✓ | ✓ | ✓ | ✓ | ✓ | ✓ |
| Fever ^c^ | ✔ | ✓ | ✓ | ✓ | ✓ | ✓ | ✓ |
| Headache | ✔ | ✓ | ✓ | ✓ | ✓ | ✓ | ✓ ^d^ |
| Sore Throat | ✔ | ✓ | ✓ | ✓ | ✓ | ✓ | ✓ ^d^ |
| **Pre-Existing Conditions and Risk Factors** | | | | | | | |
| Cardiac Disease | ✔ | ✓ | ✓ | ✓ | ✓ | ✓ | ✓ ^d^ |
| Diabetes | ✔ | ✓ | ✓ | ✓ | ✓ | ✓ | ✓ ^d^ |
| Pregnancy | ✔ | ✓ | ✓ | ✓ | ✓ | ✓ | ✓ ^d^ |
| Respiratory Disease | ✔ | ✓ | ✓ | ✓ | ✓ | ✓ | ✓ ^d^ |
| **Complications** | | | | | | | |
| Altered Mental Status | ✔ | ✓ | ✓ | ✓ | ✓ | ✓ | ✓ ^d^ |
| Encephalitis | ✔ | ✓ | ✓ | ✓ | ✓ | ✓ | ✓ ^d^ |
| ^a^ Applicable Provinces: AB, NL, NS, PEI, SK, YK.  ^b^ Significant variation in the recommended date format across case report forms: DD/MM/YYYY, MM/DD/YYYY, YYYY/MM/DD, YYYY/MMM/DD, and Unspecified.  ^c^ Minimum temperature that defines a fever has some variation between forms or is not defined.  ^d^ Only available on Québec form “QC Coronavirus COVID-19 Questionnaire D’enquête Des Cas” 2020-04-02. | | | | | | | |

Case report form data items have been generalized to a single label for this table. Table adapted from “Comparison and analysis of Canadian public health SARS-CoV-2 case report forms” [[20]](https://paperpile.com/c/VHLwzB/HO8nH).

**Table S4.** **Overview of identifier data fields found in Canadian case report forms reviewed for this analysis.**

| **Case Report Form** | **Identifier (ID)** | | |
| --- | --- | --- | --- |
|  | Personal Health Number | Case Identifiers | Other Identifiers |
| **National** ^a^ |  | **Local Case ID,**  **P/T Case ID** |  |
| **BC** | Health Care Number (Personal Health Care Number) |  | Panorama Investigation ID ^b^,  PARIS Client ID ^c^ |
| **MB** | Health Number (Personal Health Identification Number) | Case Accession Number | Additional Accession Numbers,  Investigation ID ^d^ |
| **NB** | Health Care Number  (Medicare) |  |  |
| **NWT** | Health Care Plan Number |  |  |
| **ON** |  | iPHIS Case ID | P/T Outbreak ID  Lab ID |
| **QC** | Health Insurance Card Number  (*fr.* Numéro D'assurance-Maladie) |  | V10 Code ^c,e^ (*fr*. Saisie V10),  Health Region Investigation Code (*fr.* Code RSS d’Enquête) |
| ^a^ Applicable Provinces: AB, NL, NS, PEI, SK, YK.  ^b^ [51]  ^c^ Health Care Information System  ^d^ Investigation ID of the contact’s investigation (source: MB22000-Contact-Tracing, 2020-01-13)  ^e^ Only available on Québec form “QC Coronavirus COVID-19 Questionnaire D’enquête Des Cas” 2020-04-02. | | | |

French translations are preceded by “*fr.*”. Table adapted from “Comparison and analysis of Canadian public health SARS-CoV-2 case report forms” [[20]](https://paperpile.com/c/VHLwzB/HO8nH).

**Table S5. Overview of gender data fields found in Canadian case report forms reviewed for this analysis.**

| **“Gender” Field Values** | **Case Report Form** | | | | | | |
| --- | --- | --- | --- | --- | --- | --- | --- |
|  | **National** ^a^ | **BC** | **MB** | **NB** | **NWT** | **ON** | **QC** |
| Male | **✓** | ✓ | ✓ | ✓ ^b^ | ✓ | ✓ | ✓ |
| Female | **✓** | ✓ | ✓ | ✓ ^b^ | ✓ | ✓ | ✓ |
| Intersex |  |  | ✓ |  |  |  |  |
| Other | **✓** |  |  |  |  | ✓ | ✓ ^c^ |
| Undifferentiated |  | ✓ |  |  |  |  |  |
| Unknown | **✓** | ✓ | ✓ |  |  | ✓ |  |
| X |  |  |  |  | ✓ |  |  |
| ^a^ Applicable Provinces: AB, NL, NS, PEI, SK, YK.  ^b^ Abbreviated to “M” for “Male” or “F” for “Female”  ^c^ Only available on Québec form “QC Coronavirus COVID-19 Déclaration Des Cas Confirmés Et Des Cas Cliniques De Covid-19”, 2020-04-28, version 20-210-103W. | | | | | | | |

Table adapted from “Comparison and analysis of Canadian public health SARS-CoV-2 case report forms” [[20]](https://paperpile.com/c/VHLwzB/HO8nH).

**Table S6.** **Overview of common “Host Health State/Outcome” data fields in Canadian case report forms.**

| **Common “Host Health State / Outcome”** | **Case Report Form** | | | | | | |
| --- | --- | --- | --- | --- | --- | --- | --- |
|  | **National** ^a^ | **BC** | **MB** | **NB** | **NWT** | **ON** | **QC** |
| Asymptomatic | **✓** | ✓ | ✓ |  | ✓ | ✓ | ✓ ^c^ |
| Symptomatic | ***Inferred*** | *Inferred* | ✓ | *Inferred*  ^b^ | *Inferred* | *Inferred* | *Inferred* |
| Deceased | **✓** | ✓ | ✓ |  | ✓ | ✓ | ✓ ^c^ |
| Deteriorating | **✓** |  |  |  | ✓ | ✓ | ✓ ^c^ |
| Recovered | **✓** | ✓ | ✓ |  |  | ✓ | ✓ ^c^ |
| Stable | **✓** |  |  |  | ✓ | ✓ | ✓ ^c^ |
| ^a^ Applicable Provinces: AB, NL, NS, PEI, SK, YK.  ^b^ Only appears in text in relation to health care professionals, vulnerable populations, and/or staff working in a health care setting or other setting with vulnerable populations.  ^c^ Only available on Québec form “QC Coronavirus COVID-19 Questionnaire D’enquête Des Cas” 2020-04-02. | | | | | | | |

Case report form data items have been generalized to a single label for this table. Case report forms used field labels “Host Health State” or “Host Health Outcome”, shortened to “Host Health State/Outcome” for header brevity. Values labelled “*Inferred”* if not explicitly requested in text but could theoretically be inferred from other fields (e.g., values selected under “signs & symptoms” section or “Asymptomatic” were not declared when the option is present). Table adapted from “Comparison and analysis of Canadian public health SARS-CoV-2 case report forms” [[20]](https://paperpile.com/c/VHLwzB/HO8nH).

**Table S7.** **Overview of common “Host Health Status Details” data fields in Canadian case report forms.**

| **Common “Host Health Status Details”** | **Case Report Form** | | | | | | |
| --- | --- | --- | --- | --- | --- | --- | --- |
|  | **National** ^a^ | **BC** | **MB** | **NB** | **NWT** | **ON** | **QC** ^c^ |
| Hospitalized | **✓** | ✓ | ✓ | ✓ | ✓ ^b^ | ✓ | ✓ |
| ICU | **✓** | ✓ | ✓ |  | ✓ | ✓ | ✓ |
| ICU Start Date | **✓** | ✓ | ✓ |  | ✓ | ✓ | ✓ |
| ICU End Date | **✓** | ✓ | ✓ |  |  | ✓ | ✓ |
| Mechanical Ventilation (MV) | **✓** | ✓ | ✓ |  |  | ✓ | ✓ |
| MV Start Date | **✓** |  | ✓ |  |  |  | ✓ |
| MV End Date | **✓** |  | ✓ |  |  |  | ✓ |
| Isolation | **✓** |  | ✓ |  | ✓ | ✓ | ✓ |
| Date of Death / Disposition Date | **✓** | ✓ | ✓ |  | ✓ | ✓ | ✓ |
| Death Attributed to a Respiratory Illness | **✓** |  |  |  |  | ✓ | ✓ |
| ^a^ Applicable Provinces: AB, NL, NS, PEI, SK, YK.  ^b^ Implied based on patient setting selection.  ^c^ Only available on Québec form “QC Coronavirus COVID-19 Questionnaire D’enquête Des Cas” 2020-04-02. | | | | | | | |

Case report form data items have been generalized to a single label for this table. Table adapted from “Comparison and analysis of Canadian public health SARS-CoV-2 case report forms” [[20]](https://paperpile.com/c/VHLwzB/HO8nH).

**Table S8.** **Overview of host residential information fields commonly found in the Canadian case report forms.**

| **Common**  **“Host Resident Information”** | **Case Report Form** | | | | | | |
| --- | --- | --- | --- | --- | --- | --- | --- |
|  | **National** ^a^ | **BC** | **MB** | **NB** | **NWT** | **ON** | **QC** |
| Country |  | ✓ |  |  | ✓ |  |  |
| Province/Territory | **✓** | ✓ | ✓ | ✓ |  | ✓ |  |
| City | **✓** | ✓ | ✓ | ✓ | ✓ ^b^ | ✓ | ✓ |
| Address | **✓** | ✓ | ✓ | ✓ |  | ✓ | ✓ |
| Postal Code | **✓** | ✓ | ✓ | ✓ |  | ✓ | ✓ |
| ^a^ Applicable Provinces: AB, NL, NS, PEI, SK, YK.  ^b^ Asks for “Community”. | | | | | | | |

Case report form data items have been generalized to a single label for this table. Table adapted from “Comparison and analysis of Canadian public health SARS-CoV-2 case report forms” [[20]](https://paperpile.com/c/VHLwzB/HO8nH).

**Table S9. Overview of “Signs and Symptoms” data fields commonly found in the Canadian case report forms.**

| **Common**  **“Signs & Symptoms”** | **Case Report Form** | | | | | | |
| --- | --- | --- | --- | --- | --- | --- | --- |
|  | **National** ^a^ | **BC** | **MB** | **NB** | **NWT** | **ON** | **QC** |
| Cough | **✓** | ✓ | ✓ | ✓ | ✓ | ✓ | ✓ |
| Diarrhea ^b^ | **✓** | ✓ | ✓ |  | ✓ | ✓ | ✓ ^e^ |
| Fever ^c^ | **✓** | ✓ | ✓ | ✓ | ✓ | ✓ | ✓ |
| Headache | **✓** | ✓ | ✓ | ✓ | ✓ | ✓ | ✓ ^e^ |
| Runny Nose | **✓** | ✓ | ✓ |  | ✓ | ✓ | ✓ ^e^ |
| Dyspnea | **✓** | ✓ | ✓ |  | ✓ | ✓ | ✓ |
| Sore Throat | **✓** | ✓ | ✓ | ✓ | ✓ | ✓ | ✓ ^e^ |
| Vomiting ^d^ | **✓** | ✓ | ✓ |  | ✓ | ✓ | ✓ ^e^ |
| ^a^ Applicable Provinces: AB, NL, NS, PEI, SK, YK.  ^b^ Sometimes combined with other signs or symptoms - e.g., Diarrhea/vomiting, Nausea/vomiting.  ^c^ Minimum temperature that defines a fever has some variation between forms or is not defined.  ^d^ Only available on Québec form “QC Coronavirus COVID-19 Questionnaire D’enquête Des Cas” 2020-04-02.  ^e^ In some cases available jointly as “Nausea” and “Vomiting”. | | | | | | | |

Case report form data items have been generalized to a single label for this table. Table adapted from “Comparison and analysis of Canadian public health SARS-CoV-2 case report forms” [[20]](https://paperpile.com/c/VHLwzB/HO8nH).

**Table S10. Overview of “Pre-Existing Conditions” data fields commonly found in the Canadian case report forms.**

| **Common**  **“Pre-Existing Conditions”** | **Case Report Form** | | | | | | |
| --- | --- | --- | --- | --- | --- | --- | --- |
|  | **National** ^a^ | **BC** | **MB** | **NB** | **NWT** | **ON** | **QC** ^b^ |
| Cardiac Disease | **✓** | ✓ | ✓ | ✓ | ✓ | ✓ | ✓ |
| Chronic Neurological or Neuromuscular Disorder | **✓** | ✓ | ✓ |  | ✓ | ✓ | ✓ |
| Diabetes | **✓** | ✓ | ✓ | ✓ | ✓ | ✓ | ✓ |
| Immunodeficiency | **✓** | ✓ | ✓ |  | ✓ | ✓ | ✓ |
| Liver Disease | **✓** | ✓ | ✓ |  | ✓ | ✓ | ✓ |
| Post-partum (≤6 weeks) | **✓** | ✓ |  |  | ✓ | ✓ | ✓ |
| Pregnancy | **✓** | ✓ | ✓ | ✓ | ✓ | ✓ | ✓ |
| Renal Disease | **✓** | ✓ | ✓ |  | ✓ | ✓ | ✓ |
| Respiratory Disease | **✓** | ✓ | ✓ | ✓ | ✓ | ✓ | ✓ |
| ^a^ Applicable Provinces: AB, NL, NS, PEI, SK, YK.  ^b^ Only available on Québec form “QC Coronavirus COVID-19 Questionnaire D’enquête Des Cas” 2020-04-02. | | | | | | | |

Case report form data items have been generalized to a single label for this table. Table adapted from “Comparison and analysis of Canadian public health SARS-CoV-2 case report forms” [[20]](https://paperpile.com/c/VHLwzB/HO8nH).

**Table S11. Overview of “Pre-Existing Conditions” data fields commonly found in the Canadian case report forms.**

| **Common “Complications / Clinical Evaluations”** | **Case Report Form** | | | | | | |
| --- | --- | --- | --- | --- | --- | --- | --- |
|  | **National** ^a^ | **BC** | **MB** | **NB** | **NWT** | **ON** | **QC** ^b^ |
| Altered Mental Status | **✓** | ✓ | ✓ | ✓ | ✓ | ✓ | ✓ |
| Diagnosed with Acute Respiratory Distress Syndrome | **✓** | ✓ | ✓ |  | ✓ | ✓ | ✓ |
| Encephalitis | **✓** | ✓ | ✓ | ✓ | ✓ | ✓ | ✓ |
| Renal Failure | **✓** | ✓ | ✓ |  | ✓ | ✓ | ✓ |
| Sepsis | **✓** | ✓ | ✓ |  | ✓ | ✓ | ✓ |
| ^a^ Applicable Provinces: AB, NL, NS, PEI, SK, YK.  ^b^ Only available on Québec form “QC Coronavirus COVID-19 Questionnaire D’enquête Des Cas” 2020-04-02. | | | | | | | |

Case report form data items have been generalized to a single label for this table. Table adapted from “Comparison and analysis of Canadian public health SARS-CoV-2 case report forms” [[20]](https://paperpile.com/c/VHLwzB/HO8nH).

**Table S12. Overview of “Location of Exposure fields” across Canadian case report forms.**

| **“Location of Exposure”** | **Case Report Form** | | | | | | |
| --- | --- | --- | --- | --- | --- | --- | --- |
|  | **National** ^a^ | **BC** | **MB** | **NB** | **NWT** | **ON** | **QC** ^b^ |
| Country | **✓** | *Inferred* | ✓ | *Inferred* | *Inferred* | *Inferred* | *Inferred* |
| Travel History | **✓** | ✓ | ✓ | ✓ | ✓ | ✓ | ✓ |
| Most Recent Travel | | | | | | | |
| Destination (City) | **✓** | *Inferred* | ✓ | *Inferred* | *Inferred* | ✓ | ✓ |
| Destination (State/Province/Territory) | ***Inferred*** | *Inferred* | *Inferred* | *Inferred* | *Inferred* | *Inferred* | *Inferred* |
| Destination (Country) | **✓** | ✓ | ✓ | ✓ | ✓ | ✓ | ✓ |
| Departure Date | **✓** |  | ✓ |  | ✓ | ✓ | ✓ |
| Return Date | **✓** | ✓ | ✓ | ✓ | ✓ | ✓ | ✓ |
| ^a^ Applicable Provinces: AB, NL, NS, PEI, SK, YK.  ^b^ Only available on Québec form “QC Coronavirus COVID-19 Questionnaire D’enquête Des Cas” 2020-04-02. | | | | | | | |

Case report form data items have been generalized to a single label for this table. Values labelled “*Inferred”* if not explicitly requested in text but could theoretically be inferred from other fields (e.g., if the patient hasn’t travelled one might assume exposure occurred within the region where the test occurred). Table adapted from “Comparison and analysis of Canadian public health SARS-CoV-2 case report forms” [[20]](https://paperpile.com/c/VHLwzB/HO8nH).

**Table S13. Overview of “Exposure Setting” data fields commonly found in the Canadian case report forms.**

| **Common “Exposure Settings”** | **Case Report Form** | | | | | | |
| --- | --- | --- | --- | --- | --- | --- | --- |
|  | **National** ^a^ | **BC** | **MB** | **NB** | **NWT** | **ON** | **QC** |
| Animal ^b^ | **✓** |  | ✓ |  |  |  |  |
| Conference |  | ✓ |  |  |  |  |  |
| Correctional Facility |  | ✓ | ✓ |  |  |  |  |
| Family Setting | **✓** |  |  |  |  |  | ✓^d^ |
| During Travel | **✓** ^c^ |  |  |  |  |  |  |
| Health care Setting | **✓** | ✓ | ✓ | ✓ | ✓ | ✓ | ✓ ^c^ |
| Home/Household | **✓** ^e^ | ✓ |  |  |  |  |  |
| Laboratory Setting | **✓** ^c^ | ✓ | ✓ ^c^ |  | ✓ | ✓ | ✓ |
| Long Term Care Facility |  | ✓ |  |  | ✓ | ✓ | ✓ ^d^ |
| Public Transport |  |  |  |  |  |  | ✓ ^d^ |
| School/Daycare/Etc. |  | ✓ |  | ✓ | ✓ | ✓ | ✓ |
| Shelter |  | ✓ | ✓ |  |  |  |  |
| Workplace Setting | **✓** | ✓ |  | ✓ | ✓ | ✓ | ✓ ^d^ |
| Other Residential Facility ^g^ |  | ✓ | ✓ |  |  |  | ✓ ^f^ |
| Other, specify: | **✓** | ✓ | ✓ | ✓ | ✓ |  |  |
| Unknown | **✓** | ✓ |  |  |  |  |  |
| ^a^ Applicable Provinces: AB, NL, NS, PEI, SK, YK.  ^b^ E.g., Farm, Live Animal Market, Vet.  ^c^ Inferable from other data elements.  ^d^ Only available on Québec form “QC Coronavirus COVID-19 Questionnaire D’enquête Des Cas” 2020-04-02.  ^e^ Exposure in the context of “animals” only.  ^f^ Only available on Québec form “QC Coronavirus COVID-19 Déclaration Des Cas Confirmés Et Des Cas Cliniques De Covid-19”, 2020-04-28, version 20-210-103W.  ^g^ E.g., Acute Care Facility, Assisted Living, Group Home, etc. | | | | | | | |

Case report form data items have been generalized to a single label for this table. Table adapted from “Comparison and analysis of Canadian public health SARS-CoV-2 case report forms” [[20]](https://paperpile.com/c/VHLwzB/HO8nH).

**Table S14. Overview of where “Exposure Event” information could be collected in the Canadian case report forms.**

| **Exposure Event** | **Case Report Form** | | | | | | |
| --- | --- | --- | --- | --- | --- | --- | --- |
|  | **National** ^a^ | **BC** | **MB** | **NB** | **NWT** | **ON** | **QC** |
| **Additional Information** (Not Event Specific) ^b^ | **✓** | ✓ |  | ✓ |  | ✓ |  |
| **Exposures** (General) | | | | | | | |
| **Comments** ^b^ | **✓** | ✓ |  |  |  |  |  |
| **Exposure History** | | | | | | | |
| **Details** ^b^ |  |  |  |  | ✓ |  | ✓ |
| **Exposure Settings** | | | | | | | |
| **Acquisition Event** ^b^ |  |  | ✓ |  |  |  |  |
| **Agricultural Fair or event/petting zoo** |  |  |  |  |  | ✓ ^c^ |  |
| **Conference** |  | ✓ | ✓ ^d^ |  |  |  |  |
|  | | | | | | | |
| Has opportunity to list an “Exposure Event”  ^b^ | **✓** | ✓ | ✓ | ✓ | ✓ | ✓ | ✓ |
| ^a^ Applicable Provinces: AB, NL, NS, PEI, SK, YK.  ^b^ Vocabulary not controlled.  ^c^ Only in cases with direct or indirect animal contact.  ^d^ Only for cases that have travelled in 14 days prior to symptom onset. | | | | | | | |

Case report form data items have been generalized to a single label for this table. Table adapted from “Comparison and analysis of Canadian public health SARS-CoV-2 case report forms” [[20]](https://paperpile.com/c/VHLwzB/HO8nH).

**Table S15. Overview of contact exposures data fields commonly found in the Canadian case report forms.**

| **Exposures:**  **Close, Direct, & Indirect Contact** | **Case Report Form** | | | | | | |
| --- | --- | --- | --- | --- | --- | --- | --- |
|  | **National**^a^ | **BC** | **MB** | **NB** | **NWT** | **ON** | **QC** |
| **Contact with Confirmed/Known Case** | ***Inferred*** ^b^ | *Inferred* ^2^ | ✓ ^b^ | ✓ ^b^ | ✓ | *Inferred*  ^b^ | ✓ |
| Close Contact^c^ with Confirmed/Known Case | **✓** ^b^ | ✓ ^b^ | ✓ ^b^ |  |  | ✓ ^b^ | ✓ ^b^ |
| **Contact with Probable Case** | ***Inferred*** | *Inferred* ^b^ | ✓ ^b^ |  |  | *Inferred*  ^b^ |  |
| Close Contact^c^ with Probable Case | **✓** ^b^ | ✓ ^b^ |  |  |  | ✓ ^b^ |  |
| **Close Contact^c^, Non-Household** |  |  | ✓ |  |  |  |  |
| **Contact with Someone with Similar Illness** | ***Inferred*** |  | ✓ ^b^ | *Inferred*  ^b^ |  | ✓ ^b^ | **✓ ^f,g^** |
| Close Contact^c^ with a person with a  fever or cough who has been to an affected area | **✓ ^b^** |  |  |  |  |  |  |
| Close Contact^c^ with a person with Acute Respiratory illness/group exposure |  |  |  | ✓ ^b^ |  |  |  |
| Contact with Symptomatic People |  |  |  |  |  |  | **✓ ^b,g^** |
| **Direct Patient Contact, Health care Worker** | **✓** |  |  | ✓ ^e^ |  | ✓ |  |
| **Direct Patient Contact, Health care Volunteer** | **✓** |  |  |  |  | ✓ |  |
| **Direct Customer Contact** |  |  |  |  |  |  | ✓ ^b^ |
| **Contact with Animal or Animal Products** | **✓** ^b^ |  |  |  |  | *Inferred*  ^b^ |  |
| Direct Animal Contact | **✓** ^b^ |  | ✓ ^d^ |  |  | ✓ ^b^ |  |
| Indirect Animal Contact |  |  |  |  |  | ✓ ^b^ |  |
| Direct Animal Product Contact | **✓** ^b^ |  |  |  |  | ✓ ^b^ |  |
| ^a^ Applicable Provinces: AB, NL, NS, PEI, SK, YK.  ^b^ Only for exposures that occurred within 14 days prior to symptom onset.  ^c^ Close contact is defined as a person who provided care for the patient, including health care workers, family members or other caregivers, or who had other similar close physical contact OR who lived with or otherwise had close prolonged contact with a probable or confirmed case while the case was ill [[13]](https://paperpile.com/c/VHLwzB/0vpWD). Note: Cannot guarantee all case report forms abide by this definition.  ^d^ Does not include domestic pets.  ^e^ Only if the case is symptomatic.  ^f^ Frequented environments where exposure could have occurred.  ^g^ Only available on Québec form “QC Coronavirus COVID-19 Questionnaire D’enquête Des Cas” 2020-04-02. | | | | | | | |

Case report form data items have been generalized to a single label for this table. Values labelled “*Inferred”* if not explicitly requested in text but could theoretically be inferred from other fields (e.g., “Contact” in the broadest sense can be inferred when “Close Contact” is confirmed). Table adapted from “Comparison and analysis of Canadian public health SARS-CoV-2 case report forms” [[20]](https://paperpile.com/c/VHLwzB/HO8nH).

**Table S16. Overview of “Host Role” data categories commonly found in the Canadian case report forms.**

| **Common “Host Role” Categories** | **Case Report Form** | | | | | | |
| --- | --- | --- | --- | --- | --- | --- | --- |
|  | **National** ^a^ | **BC** | **MB** | **NB** | **NWT** | **ON** | **QC** |
| Animal Handler | **Worker** |  | Worker |  |  | Worker, Visitor |  |
| Correctional Center |  | Inmate/  Resident, Patient, Worker | Resident | Patient,  Resident |  | Resident | Resident, Worker |
| Child/Day Care |  | Attendee, Worker | Attendee, Worker, Volunteer | Worker | Attendee,  Worker |  | Worker |
| Health care ^b^ | **Worker, Volunteer** | Student, Worker, Volunteer | Patient, Worker | Patient, Worker | Patient, Worker | Patient, Worker, Volunteer | Worker |
| Laboratory | **Worker** | Worker | Worker | Worker | Worker | Worker | Worker |
| Long Term / Personal Care Facility | **Resident** | Patient, Resident, Worker | Patient, Resident, Worker | Patient, Resident |  | Resident | Resident, Worker |
| School |  | Student,  Worker |  |  | Attendee,  Worker | Attendee,  Worker | Worker |
| Shelter |  | Patient,  Resident,  Worker | Resident | Patient, Resident |  |  |  |
| ^a^ Applicable Provinces: AB, NL, NS, PEI, SK, YK.  ^b^ Counted when explicit in text, but this information can be inferred from other form data. | | | | | | | |

Case report form data items have been generalized to a single label for this table. Table adapted from “Comparison and analysis of Canadian public health SARS-CoV-2 case report forms” [[20]](https://paperpile.com/c/VHLwzB/HO8nH).

# References:

*Citation number is reflective of relative position within the associated manuscript.*

23. Rhiannon Cameron, Sarah Savić-Kallesøe, Emma J Griffiths, William Hsiao. Comparison and analysis of Canadian public health SARS-CoV-2 case report forms. 2020. <https://genomecanada.ca/wp-content/uploads/2022/01/2020-12-10_crf_report_.pdf>. Accessed 22 Mar 2025.
